# The Recent Meeting of the Tex. State Med. Association

**Published:** 1887-05

**Authors:** 


### THE RECENT MEEING OF THE TEXAS MEDICAL ASSOCIATION.

A four days session of this now famous medical body was closed in this city Friday evening, April 29, ult. There were, in all, about three hundred physicians in attendance, representing all parts of this vast State. Doubtless the attendance would have been much larger but for the general depression in consequence of the protracted drought.

Much good work was done, and the forthcoming volume of Transactions, we predict, will make a creditable showing. There was a large number of papers contributed to the various sections, but unfortunately, for want of time, many were not read,—nor even announced.

At the Dallas meeting last year, the same thing occurred, and we pointed out the cause and suggested the remedy. The Association has grown to such dimensions,—the work increasing *pari passu*, that it is simply impossible to work by the schedule marked out for it in early days. We recommended doubling up the sections, with a view to economising time; that is, to have two or more sections going at once, in separate halls—as is done in the American Medical Association. We warned against a repetition of the effort to work in the old way—and the result of this last meeting has demonstrated the correctness of our position. The committee of arrangements, however, differed with us, and held that before the Association could make any departure from the custom of calling the sections in the order in which they are named in the By-Laws there would have to be made an amendment. The By-Laws say nothing about it, further than that "papers shall be read in the following sections," and there is no reason on earth why the committee of arrangements, with these two demonstrations of the failure of the old plan before them, should not provide for the next meeting a plan similar to that adopted by the American Association. On the contrary, there are many reasons why they should—the chief has been named—because, necessarily, many papers cannot be reached, and nothing is known of them till they appear in the Transactions. This is not calculated to stimulate members to prepare papers.

When one has taken the time aud trouble to prepare a paper, he is disappointed if it is not read—and chagrinned if it is not even called for, or mentioned—and it is likely that one would not be apt to put himself in a position to be similarly treated, another time. Indeed, we know of our own knowledge more than one instance where the authors of papers felt hurt and piqued, because their papers were apparently ignored—although they must have seen that it was unavoidable under the circumstances. True, all cannot come first; but it is hard to make the average member understand why *his* paper has not as good a right to be called first, as any other man's. Again, it is something of a bore to be compelled to listen to the reading of all papers—the alternative being to be absent; and if there were two or more sections in operation, one could, and would exercise a choice.

The late President pointed out in his message that the Constitution and By-Laws, made for the Association in its youth, are no longer adequate to its needs; and we trust that the committee on revision will give this feature particular attention if, in their judgment, it is necessary to legislate on the subject. For our part we hold that the division of the time and the distribution of the exercises, are matters for the local committee to determine, and we trust that our Galveston friends will take steps to obviate the difficulties which have beset the association at Dallas and at Austin. As there was not time to read all the papers, there was, necessarily, not time to discuss those that were read. Not half of those that were read were discussed, and it seems to us that that is the main object for which the association meets; it certainly is one of the most interesting as well as important features, and ample time should be allowed for a full, free and exhaustive discussion of any paper read. We surely should profit by experience.

---

Cincho Quinine is preferable in all cases where Quinine is indicated except conjestive chills, and in these it is equally as effective as Sulphate Quinine.

---

### A DAILY EDITION.

During the six days' session of the Ninth International Medical Congress THE MEDICAL REGISTER of Philadelphia will be issued daily. It will be full size, and will contain a complete report of the General Session and all of the Sections. It will at once be seen that this will offer, to those who cannot attend the Congress, a chance to have a full report of the proceedings daily. We will furnish this edition for 50 cents for the six days, postage prepaid, to any address, or any one subscribing to THE MEDICAL REGISTER for one year ($3.00 in advance), will receive the daily edition free.

Address all orders to THE MEDICAL REGISTER CO.,
1519 Walnut Street, Philadelphia, Pa.

